# DHA and EPA Content and Fatty Acid Profile of 39 Food Fishes from India

**DOI:** 10.1155/2016/4027437

**Published:** 2016-08-04

**Authors:** Bimal Prasanna Mohanty, Satabdi Ganguly, Arabinda Mahanty, T. V. Sankar, R. Anandan, Kajal Chakraborty, B. N. Paul, Debajit Sarma, J. Syama Dayal, G. Venkateshwarlu, Suseela Mathew, K. K. Asha, D. Karunakaran, Tandrima Mitra, Soumen Chanda, Neetu Shahi, Puspita Das, Partha Das, Md Shahbaz Akhtar, P. Vijayagopal, N. Sridhar

**Affiliations:** ^1^ICAR-Central Inland Fisheries Research Institute, Barrackpore, Kolkata 700120, India; ^2^ICAR-Central Institute of Fisheries Technology, Cochin 682029, India; ^3^ICAR-Central Marine Fisheries Research Institute, Cochin 682018, India; ^4^ICAR-Central Institute of Freshwater Aquaculture, Bhubaneswar 751002, India; ^5^ICAR-Directorate of Coldwater Fisheries Research, Bhimtal, Uttarakhand 263136, India; ^6^ICAR-Central Institute of Brackishwater Aquaculture, Chennai 600028, India; ^7^ICAR-Central Institute of Fisheries Education, Mumbai 400061, India

## Abstract

Docosahexaenoic acid (DHA) is the principal constituent of a variety of cells especially the brain neurons and retinal cells and plays important role in fetal brain development, development of motor skills, and visual acuity in infants, lipid metabolism, and cognitive support and along with eicosapentaenoic acid (EPA) it plays important role in preventing atherosclerosis, dementia, rheumatoid arthritis, Alzheimer's disease, and so forth. Being an essential nutrient, it is to be obtained through diet and therefore searching for affordable sources of these *ω*-3 polyunsaturated fatty acids (PUFA) is important for consumer guidance and dietary counseling. Fish is an important source of PUFA and has unique advantage that there are many food fish species available and consumers have a wide choice owing to availability and affordability. The Indian subcontinent harbors a rich fish biodiversity which markedly varies in their nutrient composition. Here we report the DHA and EPA content and fatty acid profile of 39 important food fishes (including finfishes, shellfishes, and edible molluscs from both marine water and freshwater) from India. The study showed that fishes* Tenualosa ilisha*,* Sardinella longiceps*,* Nemipterus japonicus*, and* Anabas testudineus* are rich sources of DHA and EPA. Promotion of these species as DHA rich species would enhance their utility in public health nutrition.

## 1. Introduction

Fatty acids play crucial role in maintaining health and cellular functions. The preventive effect of *ω*-3 fatty acids on coronary heart disease is based upon hundreds of experiments in animals, humans, tissue culture studies, and even clinical trials [1] which first became apparent in the investigation on the health status of Greenland Eskimos who consumed a very high fat diet from seal, whale, and fish and yet had a low incidence of coronary heart disease [2]. Further studies have shown that the kind of fat the Eskimos consumed contained large quantities of *ω*-3 fatty acids: EPA (20:5) and DHA (22:6). Moreover, deficiencies of these fatty acids lead to a host of symptoms and disorders. Among the long chain omega- (*ω*-) 3 fatty acids (LC-PUFA), docosahexaenoic acid (DHA) is the principal PUFA constituent of brain neurons, retinal cells, and primary structural component of skin, sperm, and testicles. Apart from being an important structural component of cellular membranes, it performs varieties of functions in a number of cellular processes like transport of neurotransmitters and amino acids and modulates the functioning of ion channels and responses of retinal pigments [3]. DHA has been shown to be particularly important for fetal brain development, optimal development of motor skills and visual acuity in infants, lipid metabolism in children and adults, and cognitive support in the elderly [3]. DHA along with eicosapentaenoic acid (EPA) play important role in preventing atherosclerosis, dementia, rheumatoid arthritis, diseases of old age like Alzheimer's disease (AD), and age related macular degeneration (AMD) [4–6]. Cardiovascular disease (CVD) is the leading cause of mortality in many economically developed countries and DHA plays an important role in preventing CVDs.

DHA is an essential nutrient as it is synthesized in very less quantity in human body and is obtained mainly through diet. Cold water marine fishes are the important dietary sources of DHA. Marine microalgae are the primary producers of DHA and the concentration of DHA goes on increasing up in the food chain with these microalgae at the base [7]. Diet and lifestyle issues are closely associated with a myriad of cardiovascular risk factors including abnormal plasma lipid, hypertension, insulin resistance, diabetes, and obesity, suggesting that diet based approaches may be of benefit [1]. Substantial evidence from epidemiological and clinical trial studies indicates consumption of fish; oily fish rich in long chain *ω*-fatty acids in particular reduce risk of cardiovascular mortality [1]. Low fat intake and associated chronic energy deficiency have been the major nutritional problem of developing countries. The consumption of fat has been found to be lower in developing countries, that is, 49 g/person/day in comparison to 128 g/person/day in the developed countries [8]. It has been observed that the supply of fat and *ω*-3 fatty acids decreases significantly with decreasing gross domestic product (GDP) and the total *ω*-3 fatty acid supply is below or close to the lower end of the recommended intake range in some of countries with the lower GDP [9]. Therefore, it is imperative to look for sources of PUFA, particularly DHA and EPA, and other fatty acids for steady supply for health and nutrition of millions of people in the developing countries.

Fish is an important component of human diet in most parts of the world and plays an important role as a source of health friendly fatty acids. The nutrients in fish include PUFA, especially the *ω*-3 PUFA, DHA, and EPA [10], proteins, amino acids, and micronutrients (minerals and vitamins). Besides, unlike other animal proteins, fish has the unique advantage that there are many fish species available. Fish is one of the cheapest sources of quality animal proteins and plays a great role in quenching the protein requirement in the developing and under developed countries of the world. Fish is also considered as a health food owing to its oil which is rich in PUFA [11]. The health benefits of fish oil consumption were revealed from the investigations on the Greenland Eskimos [2] and many such studies to fully explore the health benefits of fish consumption are still being carried out.

Fishes like* Salmo salar* (salmon),* Gadus morhua* (cod), and* Thunnus thynnus* (tuna) serve as the chief sources of DHA and other PUFA in the western countries. However, the Indian subcontinent harbors a rich biodiversity of fishes which markedly varies in their nutrient composition. Therefore, to fully harness the potential of different fish species for human health and nutrition, it is necessary to have comprehensive information of the fatty acid profile of different species of food fishes. In the present study, we report the *ω*-3 PUFA, DHA, and EPA content, complete fatty acid, and proximate composition of 39 important food fishes from India, which would enhance their utility in public health and nutrition.

## 2. Materials and Methods

### 2.1. Ethics Statement

The study including sample collection, experimentation, and sacrifice met the ethical guidelines, including adherence to the legal requirements of the study country. Fresh fishes were collected from the landing stations and were brought to the laboratory in ice. The study did not include any live animal. No specific permissions were required for these locations and activities as these were fish landing centers and are open for customers. The field studies did not involve endangered or protected species.

### 2.2. Collection of Samples

A total of 39 species of fishes were collected from their landing stations (Table 1). The weight of these fishes ranged between 500 and 800 g per fish except the small indigenous fishes (SIFs) and shellfishes (edible part was taken). Twelve individual fish samples were analyzed in triplicate. For the SIFs and shellfishes, three pooled samples were prepared, each sample containing up to hundred individuals. The length (cm) and weight (g) of individual fish were recorded. Scales were removed by scraping, with the edge of a knife having titanium blade, the blade was rinsed with distilled water, and fillets were removed and freed from bones. The fishes were degutted and muscle fillets were minced and kept in −40°C until usage. For small indigenous fishes, whole fishes were cleaned, descaled, and degutted, and then samples were pooled and minced and kept in −40°C preceding analysis.

### 2.3. Gross Chemical Composition

The gross chemical composition that is moisture, crude fat, crude protein, and ash contents was determined according to AOAC [12]. The minced samples were kept in an oven at 105 ± 2°C overnight until constant weight was obtained for moisture estimation. The crude protein and crude fat contents were estimated by Kjeldahl and Soxhlet methods, respectively [12]. Ash content was determined by incinerating known weight of dry sample at high temperature of 600°C for 6 h in a muffle furnace [12].

### 2.4. Lipid Extraction and Preparation of Fatty Acid Methyl Esters

Lipid extraction was carried out as per Folch et al. (1957) [13]. In brief, 30 g of minced fish samples was homogenized (using a motor pestle) in the organic solvent mixture (chloroform : methanol, 2 : 1), keeping the solvent/tissue ratio 20 : 1, and filtered applying little vacuum. The extraction and filtration procedure were repeated three times with fresh solvent mixture. The organic fractions, enriched with lipids, were collected, pooled, and dried in a rotary evaporator. The dried lipids were weighed, dissolved in chloroform, and stored in graduated test tubes at 4°C. Fatty acid methyl esters (FAME) were prepared from the extracted fat as per Metcalfe et al. (1966) [14].

### 2.5. Fatty Acid Analysis

Fatty acid compositions (oils) of the samples were determined by Gas Chromatography-Ion Trap Mass Spectrometry (GC/IT-MS), Thermo Scientific ITQ 900. Briefly, the FAME was analyzed by injecting 1 *μ*L (30 : 1 split ratio) into GC-MS. The fatty acids were identified and quantified using a GC (Trace GC Ultra, Thermo Scientific) equipped with a capillary column (TR-FAME, 30 m × 0.25 mm, 0.25 *μ*m film thickness) and an MS (ITQ 900, Thermo Scientific) attached to it. For separation of fatty acids, the oven temperature program was set as follows: 1 min initial hold at 50°C, temperature raised from 50 to 150°C at the rate of 20°C per min followed by a hold of 15 min at 150°C, temperature raised from 150 to 240°C at the rate of 20°C per min, and a final hold of 2 min at 240°C. Helium was used as a carrier gas with column flow rate of 1.0 mL per min. The transfer line and ion source temperatures were 250 and 220°C, respectively. The MS conditions were as follows: ionization voltage: 70 eV, range of 40–500* m/z*, and the scan time equal to the GC run time. The individual constituents showed by GC were identified and quantified by comparing the retention times and peak areas to those of standards (ME-14-KT and ME-19-KT, SUPELCO Analytical) and by using the NIST Library (version 2.0, 2008).

### 2.6. Statistical Methods

All the data are reported as mean ± standard deviation. One-way ANOVA was also employed to compare the variation in fatty acid with respect to different species (*p* < 0.05).

## 3. Results

In the present study, 39 food fishes were selected from different habitats which include 12 in marine water, 3 in brackish water, 14 in freshwater, and 5 in cold water and 3 prawns and 2 mussels considering their commercial importance and consumer preference. Moisture, crude protein, crude fat, and ash contents of the muscle tissue of these fish species are shown in Table 1. The crude fat content showed that, among the species studied, the migratory fish* T. ilisha* contains the highest amount of fat (10.5%) followed by the marine fish* S. longiceps* (9.2%) (Table 1). The fish species studied have been further categorized into different groups as lean fish, low fat, medium fat, and high fat according to the fat content [9] (Table 2). The overview of fatty acid composition of fishes from different habitats is discussed in the following sections (Tables 3 [Table tab4] [Table tab5] [Table tab6] [Table tab7]–8).

### 3.1. Overview of Fat Content and Fatty Acid Composition

The fat contents of the fishes varied markedly among the species (0.6–10.5%).* T. ilisha* was found to contain the highest amount of fat with 42.8% saturated fatty acids (SFA), 30.6% monounsaturated fatty acids (MUFA), and 22.0% PUFA [15] and myristic acid (C14:0) was the predominant fatty acid (37.8%) in* T. ilisha*.* S. longiceps*,* A. testudineus*, and* G. chapra* were found to be among the other fat rich fishes and palmitic acid was found to be major fatty acid in these fishes (Table 3). The Indian major carps* C. catla*,* L. rohita*, and* C. mrigala* are the major freshwater fishes cultured across the country; they were found to be containing 2.8, 2.7, and 2.8% fat, respectively (Table 1). The fat content in the SIFs was 6.9% in* A. testudineus*, 4.3% in* A. mola*, and 4.9% in* P. sophore*, respectively [16]. Majority of the fatty acids in the SIFs are monounsaturated fatty acids (MUFA) like oleic acid and linolenic acid.* A. mola* and* P. sophore* were found to be rich in MUFA whereas* A. testudineus* was rich in SFA (Table 6).

### 3.2. Overview of DHA and EPA Profile

**Figure 1:**
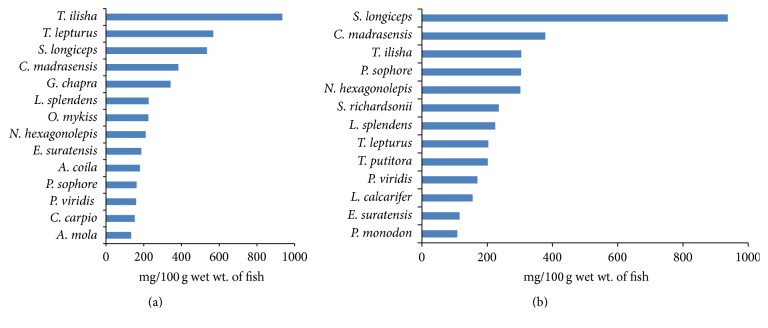
DHA (a) and EPA (b) content of important Indian food fishes from India.

*T. ilisha* was found to contain the highest amount of DHA followed by* T. lepturus*. Similarly EPA content was highest in* S. longiceps* followed by* C. madrasensis*. Among the cold water fishes* N. hexagonolepis* and* O. mykiss* were rich in DHA while* S. richardsonii* and* N. hexagonolepis* were rich in EPA. Among SIFs,* G. chapra* was found to be rich in DHA and* P. sophore* in EPA (Table 9 and Figure 1).

### 3.3. *ω*-3, *ω*-6 Fatty Acids, and Their Ratio

The *ω*-3 and *ω*-6 fatty acid content varied between 12.3 and 43.55% and 1.92 and 34.12% of total fat in the fishes and shellfishes studied (Tables 3 [Table tab4] [Table tab5] [Table tab6] [Table tab7]–8). The *ω*-3 contents were high in the fishes* S. longiceps* (21.40%),* T. ilisha* (14.2%),* R. kanagurta* (34.12%),* N. japonicas* (33.7%),* C. catla* (22.7%), and* P. sophore* (27.9%). The *ω*-6 contents were high in* L. calcarifer* (12.1%),* T. albacares* (15.6%),* A. mola* (12.7%), and* P. sophore* (15.6%).

The *ω*-3/*ω*-6 ratio was 4.32, 4.82, and 4.66 in* S. longiceps*,* T. lepturus*, and* H. neherus*, respectively.* T. ilisha* is one of the most oily fishes which had a *ω*-3/*ω*-6 ratio of 2.26. The ratio was found to be more than 2 in the brackish water fish* M. cephalus*, freshwater fish* C. catla*,* A. testudineus*, the cold water fish* S. richardsonii*, and the shellfish* P. viridis*.* A. testudineus* was found to be containing 7.9 times more *ω*-3 than *ω*-6 fatty acid and* S. richardsonii* and* P. viridis* contained 5 times more *ω*-3 than *ω*-6 fatty acid. The *ω*-3/*ω*-6 ratio was found to be more than 1 in majority of fishes except* T. albacares* (0.7),* L. rohita* (0.5),* C. mrigala* (0.21),* S. seenghala* (0.8),* A. mola* (0.9),* O. mykiss* (0.9), and* M. rosenbergii* (0.49) [16, 17].

## 4. Discussion

Fish, shellfish, and sea mammals are rich source of DHA and EPA [18]. The tropical countries including India are rich in fish biodiversity. There are wide varieties of fishes available which could provide good amount of PUFA; however, such information on many fish species is not well documented. Here we report the PUFA content, notably DHA and EPA content, and complete fatty acid composition as well as proximate composition of 39 food fishes from India (Tables 1 [Table tab2] [Table tab3] [Table tab4] [Table tab5] [Table tab6] [Table tab7]–8) which could be useful in dietary recommendations and in clinical nutrition.

The *ω*-3 fatty acids, DHA and EPA, are essential nutrients that enhance quality of life and lower the risk of premature death. DHA is proven to be essential to pre- and postnatal brain development whereas EPA seems more influential on behavior and mood [3]. The DHA in combination with EPA is prescribed for a variety of clinical conditions, including the prevention and reversal of heart disease, asthma, cancer, lung diseases, systemic lupus erythematosus (SLE), high cholesterol, high blood pressure, psoriasis, rheumatoid arthritis, bipolar disorder, certain inflammations of the digestive system (ulcerative colitis), and preventing migraine pain [19]. Supplementation of EPA and DHA is also prescribed during pregnancy as these have got many crucial roles in critical periods of growth of the fetus and also protect them further from the onset of metabolic diseases in adulthood [20, 21]. Lack of these fatty acids is also considered a leading cause of attention deficit hyperactivity disorder (ADHD), a neurobehavioral disorder that is defined by persistent symptoms of hyperactivity/impulsivity and inattention most commonly seen in childhood and adolescence, which often extend to the adult years [22, 23]. These fatty acids are manufactured from natural fish/vegetable oils rich in PUFA and distributed under different pharmaceutical companies under different trade names. The 2010 US Dietary Guidelines recommend that individuals at both higher and average CVD risks should consume an average of at least 250 mg/day EPA + DHA (1,750 mg/week) [18]. As fishes like* T. ilisha*,* S. longiceps*,* S. richardsonii*, and* N. hexagonolepis* are very rich in EPA and DHA, these fishes can serve as natural dietary supplements for both EPA and DHA in all the above stated clinical conditions. Further, it has been surveyed that fish oil supplements offer the lowest cost of EPA and DHA [24]. Therefore, taking into consideration the pleiotropic nature of their actions, that dietary supplementation with this long chain PUFA would lead to improvements in overall health parameters.

The hilsa shad* Tenualosa ilisha* (hilsa) is a commercially important food fish in India, Bangladesh, and the adjoining countries and is long known to be rich in oil, unlike many of its estuarine and freshwater counterparts [15, 25].* T. ilisha* enjoys high consumer preference, owing to its taste, flavor, and other culinary properties. Myristic acid, which is used as a common flavoring agent in food items [26], was found to be the predominant fatty acid in* T. ilisha*. The flavor of* T. ilisha* could be due to its high myristic acid content. Atlantic salmon (*Salmo salar*) is considered among the best animal proteins due to its easy digestibility and absorption and it is popular for its fat content rich in important fatty acids and triglycerides [27]. In comparison with salmon,* T. ilisha* was found to be containing more amounts of fat and *ω*-6 fatty acids (7.8%). Although the *ω*-3 content was much lower, the total EPA and DHA content was closer to that of salmon [27]. The protein content of* T. ilisha* (20.7%) is also comparable to that of salmon (18.8%) [28, 29]. Considering these facts, the significance of* T. ilisha* in health and nutrition is comparable to salmon. Due to its high food and nutritional value,* T. ilisha* is a potent species which is being tried for domestication and aquaculture.

Along with* T. ilisha*, other fishes like* S. longiceps* and* N. japonicus* are rich sources of both EPA and DHA. The SIFs are considered as rich sources of micronutrients (vitamins and minerals); however, in the present investigation, it was found that the SIFs* G. chapra* and* P. sophore* [16] contain fair amount of DHA and EPA and these fishes can be recommended as dietary sources of DHA and EPA where other sources are not available.

## 5. Conclusion

In conclusion, the present study has generated important food data on the DHA, EPA, and fatty acid profile of important food fishes from India. The information generated is being catalogued in the database (http://www.cifri.res.in/outreach/). The information generated would be useful in formulation of dietary guidelines and it could be useful for a vast majority of population including 1.27 billion from India alone, which account for 17.5% of the world population. The species like* T. ilisha*,* S. longiceps*,* N. japonicus*, and* A. testudineus* are found to be rich sources of both the *ω*-3 PUFA, DHA, and EPA and could be regarded as the subcontinental counterparts of salmon, mackerel, and tuna, the DHA rich fishes of the western world. Mass awareness campaigns to popularize these fishes as rich source of DHA are necessary for harnessing the important therapeutic value of these fishes in community nutrition and these fishes can also be prescribed under specific clinical conditions originating due to DHA and EPA deficiency, thus increasing their utility in clinical nutrition.

## Figures and Tables

**Table 1:** Proximate composition of thirty-nine important food fishes from India.

Species	Habitat	Moisture (% ww)	Crude protein (% ww)	Crude fat (% ww)	Ash (% ww)
*Ailia coila*	Freshwater (captured)	82.8 ± 0.2	12.9 ± 0.5	1.8 ± 0.0	2.0 ± 0.0
*Amblypharyngodon mola*	Freshwater (captured)	76.2 ± 1.1	16.3 ± 0.8^1^	4.3 ± 0.0	4.0 ± 0.9
*Anabas testudineus*	Freshwater (captured)	68.0 ± 0.7	16.9 ± 0.5^1^	6.9 ± 0.6	5.3 ± 0.2
*Catla catla*	Freshwater (aquacultured)	76.2 ± 0.3	16.2 ± 0.5^1^	2.8 ± 0.3	2.5 ± 0.1
*Cirrhinus mrigala*	Freshwater (aquacultured)	75.3 ± 0.6	15.5 ± 0.5^1^	2.8 ± 0.3	2.5 ± 0.1
*Clarias batrachus*	Freshwater (captured)	75.9 ± 0.7	16.4 ± 0.3^1^	3.7 ± 0.4	2.3 ± 0.0
*Crassostrea madrasensis*	Marine water (captured)	80.1 ± 0.7	16.8 ± 0.1^1^	2.7 ± 0.2	1.3 ± 0.1
*Cyprinus carpio*	Cold water (captured)	77.2 ± 0.3	17.9 ± 0.8^1^	3.0 ± 0.0	1.3 ± 0.1
*Epinephelus *spp.	Marine water (captured)	78.5 ± 1.5	18.1 ± 1.1^1^	0.9 ± 0.5	1.5 ± 0.5
*Etroplus suratensis*	Brackish water (captured)	74.2 ± 0.5	20.4 ± 0.8	4.7 ± 0.8	1.4 ± 0.1
*Euthynnus affinis*	Marine water (captured)	75.7 ± 0.1	20.9 ± 0.1	1.9 ± 0.0	1.5 ± 0.0
*Fenneropenaeus indicus*	Brackish water (captured)	82.2 ± 0.9	16.4 ± 0.3	0.7 ± 0.4	1.4 ± 0.1
*Gudusia chapra*	Freshwater (captured)	76.7 ± 0.3	14.1 ± 0.1	5.7 ± 0.0	2.9 ± 0.0
*Harpadon nehereus*	Marine water (captured)	87.5 ± 2.0	8.2 ± 0.9	2.2 ± 0.2	1.1 ± 0.2
*Heteropneustes fossilis*	Freshwater (captured)	76.7 ± 1.1	16.3 ± 0.4^1^	2.7 ± 0.5	2.6 ± 0.1
*Katsuwonus pelamis*	Marine water (captured)	70.6 ± 7.4	22.4 ± 2.9^1^	1.2 ± 1.1	1.9 ± 0.8
*Labeo rohita*	Freshwater (aquacultured)	75.6 ± 0.5	15.9 ± 0.4^1^	2.7 ± 0.2	2.6 ± 0.2
*Lates calcarifer*	Brackish water (captured)	72.8 ± 0.6	21.1 ± 0.9	2.6 ± 0.5	1.6 ± 0.1
*Leiognathus splendens*	Marine water (captured)	74.7 ± 3.7	17.2 ± 1.6^1^	3.8 ± 3.7	3.1 ± 0.7
*Macrobrachium rosenbergii*	Marine water (captured)	73.5 ± 0.6	16.9 ± 0.4	4.4 ± 0.2	4.9 ± 0.2
*Mugil cephalus*	Brackish water (captured)	75.6 ± 0.6	20.0 ± 0.9	3.3 ± 0.7	1.3 ± 0.1
*Nemipterus japonicus*	Marine water (captured)	78.5 ± 0.1	15.4 ± 0.2^1^	5.1 ± 0.0	1.0 ± 0.0
*Neolissochilus hexagonolepis*	Cold water (captured)	75.3 ± 0.1	18.2 ± 0.3^1^	3.3 ± 0.1	1.4 ± 0.0
*Oncorhynchus mykiss*	Cold water (captured)	74.7 ± 0.3	17.9 ± 0.0^1^	3.8 ± 0.1	1.8 ± 0.0
*Penaeus monodon*	Brackish water (captured)	76.3 ± 0.5	19.4 ± 0.2	0.7 ± 0.2	3.1 ± 0.1
*Perna viridis*	Marine water (captured)	83.5 ± 0.5	11.0 ± 0.1^1^	1.7 ± 0.0	1.4 ± 0.0
*Puntius sophore*	Freshwater (captured)	75.7 ± 1.9	16.3 ± 0.9^1^	4.9 ± 0.5	3.4 ± 0.1
*Rastrelliger kanagurta*	Marine water (captured)	78.2 ± 0.1	19.2 ± 0.1^1^	1.7 ± 0.0	1.2 ± 0.0
*Rita rita*	Freshwater (captured)	77.8 ± 4.3	19.5 ± 1.2	1.6 ± 0.0	1.0 ± 0.1
*Sardinella longiceps*	Marine water (captured)	71.3 ± 7.1	17.1 ± 1.4^1^	9.2 ± 5.8	2.3 ± 0.6
*Schizothorax richardsonii*	Cold water (captured)	77.3 ± 0.0	16.4 ± 0.1^1^	2.5 ± 0.0	1.2 ± 0.0
*Sperata seenghala*	Freshwater (captured)	79.4 ± 1.2	19.0 ± 1.3^1^	0.8 ± 0.4	0.9 ± 0.2
*Stolephorus commersonii*	Marine water (captured)	79.4 ± 0.1	16.4 ± 0.1^1^	1.2 ± 0.0	3.2 ± 0.2
*Stolephorus waitei*	Marine water (captured)	79.9 ± 0.1	20.3 ± 0.1^1^	1.1 ± 0.0	3.3 ± 0.3
*Tenualosa ilisha*	Freshwater (captured)	66.9 ± 4.2	20.7 ± 2.7^1^	10.5 ± 4.6	1.1 ± 0.5
*Thunnus albacares*	Marine water (captured)	74.1 ± 0.1	23.9 ± 0.1^1^	0.6 ± 0.0	1.4 ± 0.0
*Tor putitora*	Cold water (captured)	74.9 ± 0.1	17.9 ± 0.5^1^	4.3 ± 0.1	1.5 ± 0.1
*Trichiurus lepturus*	Marine water (captured)	75.5 ± 3.6	17.9 ± 1.5^1^	3.4 ± 4.1	1.6 ± 0.4
*Xenentodon cancila*	Freshwater (captured)	78.2 ± 0.7	15.7 ± 0.3	0.7 ± 0.0	3.6 ± 0.1

^1^Data previously published by Mohanty et al. [28].

Values are reported as mean ± standard deviation.

**Table 2 tab2:** Classification of 39 Indian food fish species due to fat content.

Classification	Samples
Lean meat (<2% fat)	*Euthynnus affinis* *Ailia coila* *Perna viridis* *Rastrelliger kanagurta* *Rita rita* *Katsuwonus pelamis* *Stolephorus commersonii* *Stolephorus waitei* *Epinephelus *spp. *Sperata seenghala* *Fenneropenaeus indicus* *Penaeus monodon* *Xenentodon cancila* *Thunnus albacares*

Low fat fish (2–4% fat)	*Leiognathus splendens* *Oncorhynchus mykiss* *Clarias batrachus* *Trichiurus lepturus* *Mugil cephalus* *Neolissochilus hexagonolepis* *Cyprinus carpio* *Catla catla* *Cirrhinus mrigala* *Crassostrea madrasensis* *Heteropneustes fossilis* *Labeo rohita* *Lates calcarifer* *Schizothorax richardsonii* *Harpadon nehereus*

Medium fat fish (4–8% fat)	*Anabas testudineus* *Gudusia chapra* *Nemipterus japonicas* *Puntius sophore* *Etroplus suratensis* *Macrobrachium rosenbergii* *Amblypharyngodon mola* *Tor putitora*

High fat fish (>8%)	*Tenualosa ilisha* *Sardinella longiceps*

**Table 3 tab3:** Fatty acid composition of important marine fishes.

Fatty acids (%)	*T. albacares* ^1^	*E. affinis*	*S. waitei*	*S. commersonii* ^1^	*R. kanagurta* ^1^	*N. japonicus* ^1^	*S. longiceps*	*K. pelamis*	*Epinephelus *spp.	*L. splendens*	*T. lepturus*	*H. nehereus*
*Saturated fatty acids (SFA)*												
C4:0–C11:0	*—*	*—*	*—*	*—*	*—*	*—*	*—*	*—*	*—*	*—*	*—*	*—*
C6:0	*—*	*—*	*—*	*—*	*—*	*—*	*—*	*—*	*—*	*—*	*—*	0.0 ± 0.0
C8:0	*—*	*—*	*—*	*—*	*—*	*—*	*—*	*—*	*—*	*—*	*—*	0.3 ± 0.0
C12:0	0.4 ± 0.1^a^	2.6 ± 0.5^b^	0.1 ± 0.0^c^	0.8 ± 0.0^d^	0.1 ± 0.0^c^	*—*	0.2 ± 0.0^e^	0.18 ± 0.1^f^	0.4 ± 0.1^g^	0.3 ± 0.0^g^	0.1 ± 0.0^f^	0.3 ± 0.0^g^
C13:0	1.3 ± 0.0^a^	*—*	1.7 ± 0.6^a^	7.2 ± 2.4^b^	1.5 ± 0.3^a^	0.1 ± 0.0^c^	*—*	*—*	*—*	*—*	*—*	0.0 ± 0.0^d^
C14:0	2.1 ± 0.3^a^	*—*	0.9 ± 0.1^b^	6.8 ± 1.9^c^	1.2 ± 0.6^d^	1.2 ± 0.3^d^	9.8 ± 1.2^e^	2.6 ± 0.1^a^	4.3 ± 1.2^f^	4.0 ± 1.1^f^	5.3 ± 1.2^g^	4.4 ± 1.2^f^
C15:0	0.9 ± 0.0^a^	1.7 ± 0.5^b^	0.5 ± 0.0^a^	1.3 ± 0.4^b^	0.7 ± 0.1^a^	1.1 ± 0.6^b^	0.7 ± 0.1^a^	0.8 ± 0.2^a^	1.0 ± 0.2^c^	1.8 ± 0.3^d^	0.8 ± 0.0^a^	1.0 ± 0.5^c^
C16:0	31.6 ± 8.9^a^	32.2 ± 9.6^b^	23.8 ± 5.6^c^	41.3 ± 9.6^d^	22.1 ± 9.6^e^	23.0 ± 8.8^f^	20.1 ± 2.3^g^	24.1 ± 1.9^c^	27.0 ± 2.9^h^	23.3 ± 2.8^f^	24.4 ± 3.2^c^	17.3 ± 3.6^i^
C17:0	1.5 ± 0.3^a^	0.9 ± 0.1^b^	0.8 ± 0.1^b^	1.5 ± 0.6^a^	1.2 ± 0.3^c^	1.5 ± 0.3^a^	0.7 ± 0.2^d^	1.2 ± 0.0^c^	0.8 ± 0.2^b^	1.4 ± 0.2^e^	0.9 ± 0.1^b^	1.1 ± 0.3^f^
C18:0	12.9 ± 5.6^a^	17.6 ± 5.6^b^	13.2 ± 6.6^c^	13.3 ± 3.6^c^	12.9 ± 5.6^a^	13.3 ± 4.5^c^	6.0 ± 1.3^d^	11.2 ± 2.6^e^	10.1 ± 1.7^f^	10.4 ± 2.3^f^	9.5 ± 1.2^g^	9.8 ± 2.6^g^
C19:0	*—*	8.9 ± 2.6^a^	3.2 ± 0.8^b^	*—*	*—*	*—*	*—*	*—*	*—*	*—*	*—*	0.4 ± 0.0^c^
C20:0	*—*	*—*	*—*	*—*	*—*	*—*	0.7 ± 0.1^a^	0.5 ± 0.0^b^	0.6 ± 0.1^c^	0.7 ± 0.2^c^	0.6 ± 0.0^b^	0.6 ± 0.0^b^
C22:0	*—*	*—*	*—*	*—*	*—*	*—*	0.3 ± 0.1^a^	0.2 ± 0.0	0.4 ± 0.1	0.4 ± 0.0	0.3 ± 0.0	*—*
C23:0	*—*	*—*	*—*	*—*	*—*	*—*	*—*	*—*	*—*	*—*	*—*	0.3 ± 0.0
C24:0	*—*	*—*	*—*	*—*	*—*	*—*	0.8 ± 0.1^a^	0.3 ± 0.0^b^	0.4 ± 0.0^c^	0.5 ± 0.1^d^	0.1 ± 0.0^e^	*—*

∑SFA	50.9	63.8	44.3	72.3	39.9	40.2	39.40	41.3	45.2	42.8	42.1	36.4

*Monounsaturated fatty acids (MUFA)*												
C14:1	—	—	—	—	—	—	0.2 ± 0.0^a^	0.1 ± 0.0^b^	0.4 ± 0.1^c^	0.2 ± 0.0^a^	0.1 ± 0.0^b^	—
C15:1	—	—	—	—	—	—	0.1 ± 0.0^a^	0.1 ± 0.0^b^	0.3 ± 0.1^c^	0.1 ± 0.0^a^	0.1 ± 0.0^d^	—
C16:1	2.9 ± 0.6^a^	3.7 ± 0.9^b^	2.1 ± 0.8^c^	6.2 ± 1.9^d^	2.2 ± 0.6^c^	3.4 ± 1.3^b^	9.0 ± 1.3^e^	3.3 ± 0.1^f^	6.4 ± 2.1^g^	5.9 ± 1.2^h^	5.9 ± 1.3^h^	8.3 ± 1.6^i^
C17:1	0.3 ± 0.0^a^	—	0.4 ± 0.0^b^	—	0.3 ± 0.0^a^	0.2 ± 0.0^c^	—	—	—	—	—	—
C18:1	13.8 ± 7.8^a^	11.4 ± 2.3^b^	11.0 ± 5.9^b^	8.4 ± 3.6^c^	15.1 ± 4.6^d^	14.2 ± 6.5^e^	11.5 ± 2.3^b^	14.1 ± 2.3^e^	16.2 ± 3.6^f^	14.4 ± 3.1^a^	19.5 ± 3.2^g^	14.6 ± 3.2^e^
C20:1	0.9 ± 0.2^a^	—	0.5 ± 0.0^b^	—	0.9 ± 0.1^a^	—	0.4 ± 0.0^c^	0.5 ± 0.0^c^	—	0.5 ± 0.0^d^	0.5 ± 0.2^c^	1.1 ± 0.5^e^
C22:1	0.3 ± 0.0^a^	0.8 ± 0.1^b^	0.2 ± 0.0^c^	—	1.5 ± 0.0^d^	1.7 ± 0.1^d^	2.1 ± 0.9^e^	3.2 ± 0.9^f^	3.1 ± 1.2^g^	3.0 ± 1.1^g^	1.8 ± 0.9^d^	0.1 ± 0.0^h^
C24:1	2.4 ± 0.1^a^	2.1 ± 0.2^a^	1.6 ± 0.3^b^	—	—	—	0.7 ± 0.1^c^	0.3 ± 0.0^d^	0.3 ± 0.0^e^	1.1 ± 0.2^f^	0.6 ± 0.1^g^	—

∑MUFA	20.5	18.1	15.7	14.6	19.9	19.5	24.1	21.6	26.9	25.3	28.7	24.1

*Polyunsaturated fatty acids (PUFA)*												
C16:2 *ω*-4	—	—	—	—	—	—	0.2 ± 0.0^a^	0.3 ± 0.0^b^	0.4 ± 0.0^c^	0.3 ± 0.0^a^	0.2 ± 0.0^b^	—
C16:3 *ω*-4	—	—	—	—	—	—	0.2 ± 0.0^a^	0.1 ± 0.0^b^	0.1 ± 0.0^b^	0.2 ± 0.0^a^	0.2 ± 0.0^a^	—
C18:2 *ω*-6	13.0 ± 1.2^a^	0.9 ± 0.1^b^	0.4 ± 0.0^c^	1.9 ± 0.0^d^	0.1 ± 0.0^e^	—	1.8 ± 0.1^f^	1.4 ± 0.9^g^	1.6 ± 0.0^g^	1.7 ± 0.9^f^	1.3 ± 0.2^h^	1.0 ± 0.5^i^
C18:3 *ω*-3	1.4 ± 0.1^a^	0.9 ± 0.1^b^	1.2 ± 0.6^a^	1.6 ± 0.0^b^	0.4 ± 0.0^b^	0.6 ± 0.4^b^	0.5 ± 0.1^c^	0.8 ± 0.1^c^	0.7 ± 0.1^c^	1.2 ± 0.1^d^	0.4 ± 0.0^e^	0.4 ± 0.0^e^
C18:3 *ω*-6	—	—	—	—	—	—	0.7 ± 0.1^a^	1.3 ± 0.9^b^	1.0 ± 0.2^b^	1.9 ± 0.3^c^	0.8 ± 0.2^d^	—
C18:4 *ω*-3	—	—	—	—	—	—	0.1 ± 0.0^a^	—	0.1 ± 0.0^a^	0.1 ± 0.0^a^	0.0 ± 0.0^b^	0.2 ± 0.0^a^
C20:2 *ω*-6	1.0 ± 0.0^a^	1.2 ± 0.3^b^	1.6 ± 0.5^b^	—	0.7 ± 0.0^c^	—	1.1 ± 0.2^a^	0.7 ± 0.1^c^	0.9 ± 0.1^d^	0.6 ± 0.1^e^	0.4 ± 0.1^f^	0.3 ± 0.1^g^
C20:3 *ω*-6	1.1 ± 0.0^a^	0.7 ± 0.1^b^	0.2 ± 0.0^c^	—	2.2 ± 0.0^d^	1.8 ± 0.0^e^	0.2 ± 0.0^c^	0.4 ± 0.0^f^	0.3 ± 0.0^g^	0.3 ± 0.0^g^	0.3 ± 0.0^g^	0.2 ± 0.0^h^
C20:3 *ω*-3	—	—	—	—	—	—	—	—	—	—	—	0.2 ± 0.0
C20:4 *ω*-6	0.5 ± 0.3^a^	1.0 ± 0.1^b^	7.3 ± 2.6^c^	2.1 ± 0.9^d^	2.9 ± 0.9^e^	4.2 ± 1.3^f^	1.1 ± 0.1^b^	—	1.3 ± 0.2^b^	0.9 ± 0.1^h^	1.1 ± 0.2^b^	5.0 ± 1.1^i^
C20:5 *ω*-3 (EPA)	3.0 ± 0.2^a^	3.0 ± 0.5^a^	5.6 ± 1.3^b^	1.6 ± 0.3^c^	5.2 ± 1.2^d^	6.6 ± 1.3^e^	12.3 ± 1.3^f^	5.1 ± 1.9^d^	3.6 ± 1.1^g^	7.1 ± 1.2^h^	4.4 ± 1.0^i^	7.9 ± 2.3^h^
C22:5 *ω*-3	—	—	—	—	—	—	1.3 ± 0.2^a^	2.0 ± 0.5^b^	1.3 ± 0.3^a^	1.3 ± 0.4^a^	1.6 ± 0.2^c^	2.2 ± 0.9^d^
C22:6 *ω*-3 (DHA)	8.3 ± 2.3^a^	5.0 ± 1.6^b^	23.2 ± 8.7^c^	5.8 ± 1.9^b^	28.5 ± 5.6^d^	26.5 ± 1.6^e^	6.9 ± 1.5^f^	—	8.2 ± 2.1^g^	7.2 ± 1.3^h^	12.2 ± 2.9^i^	20.3 ± 3.3^j^

∑PUFA	28.4	12.7	39.5	13.1	40.1	39.8	26.8	12.3	19.7	23.1	23.3	38.1

∑*ω*-3	12.7	8.0	30.0	9.0	34.1	33.7	21.4	7.9	14.0	16.9	18.8	31.3
∑*ω*-6	15.6	1.9	9.5	4.0	5.9	6.0	4.9	3.9	5.0	5.6	3.91	6.7
*ω*-3/*ω*-6	0.8	4.3	3.2	2.2	5.7	5.6	4.3	2.03	2.7	3.0	4.8	4.6

^1^Data previously published by Aneesh et al. [30].

Values are reported as mean ± standard deviation.

Values in rows sharing same superscripts are not statistically different (*p* < 0.05).

—, not detected.

EPA: eicosapentaenoic acid; DHA: docosahexaenoic acid.

**Table 4 tab4:** Fatty acid composition of important brackish water fishes.

Fatty acids (%)	*L. calcarifer*	*M. cephalus*	*E. suratensis*
*Saturated fatty acids (SFA)*			
C4:0–C11:0	—	—	—
C12:0	0.2 ± 0.2^a^	0.0 ± 0.0^b^	0.1 ± 0.0^a^
C13:0	—	—	—
C14:0	5.6 ± 0.6^a^	5.8 ± 0.8^a^	5.6 ± 0.7^a^
C15:0	0.7 ± 0.3^a^	1.2 ± 0.3^b^	0.6 ± 0.2^a^
C16:0	20.1 ± 1.7^a^	20.4 ± 1.8^a^	19.9 ± 1.4^a^
C17:0	0.5 ± 0.1^a^	0.8 ± 0.4^b^	0.6 ± 0.0^c^
C18:0	6.1 ± 0.4^a^	3.6 ± 0.4^b^	6.1 ± 0.7^a^
C20:0	0.4 ± 0.2^a^	0.2 ± 0.1^b^	0.5 ± 0.2^a^
C21:0	1.0 ± 0.6^a^	0.1 ± 0.1^b^	1.1 ± 0.5^a^
C22:0	0.3 ± 0.2^a^	0.2 ± 0.1^a^	0.6 ± 0.2^b^
C23:0	0.1 ± 0.0^a^	—	0.1 ± 0.1^b^
C24:0	0.4 ± 0.2^a^	—	—

∑SFA	35.6	32.5	35.7

*Monounsaturated fatty acids (MUFA)*			
C14:1	0.0 ± 0.0^a^	0.1 ± 0.0^b^	0.0 ± 0.0^a^
C16:1	7.6 ± 0.7^a^	9.9 ± 0.9^b^	7.7 ± 0.7^a^
C17:1	0.4 ± 0.3^a^	0.4 ± 0.2^a^	0.3 ± 0.2^b^
C18:1	18.5 ± 2.2^a^	8.2 ± 0.6^b^	16.0 ± 1.1^c^
C20:1	0.6 ± 0.2^a^	0.3 ± 0.1^b^	0.6 ± 0.1^a^
C22:1	0.3 ± 0.5^a^	0.4 ± 0.5^b^	0.4 ± 0.2^b^
C24:1	0.1 ± 0.1^a^	0.1 ± 0.0^b^	0.2 ± 0.1^c^

∑MUFA	27.8	19.7	25.6

*Polyunsaturated fatty acids (PUFA)*			
C18:2 *ω*-6	9.7 ± 1.5^a^	1.7 ± 0.5^b^	7.2 ± 1.0^c^
C18:3 *ω*-3	1.9 ± 0.1^a^	1.2 ± 0.8^b^	1.0 ± 0.1^b^
C18:3 *ω*-6	0.4 ± 0.3^a^	0.5 ± 0.1^b^	0.6 ± 0.2^c^
C20:2 *ω*-6	0.2 ± 0.1^a^	0.2 ± 0.8^a^	0.3 ± 0.3^b^
C20:3 *ω*-6	0.5 ± 0.2^a^	0.2 ± 0.2^b^	0.5 ± 0.1^c^
C20:3 *ω*-3	0.0 ± 0.0^a^	—	—
C20:4 *ω*-6	1.2 ± 0.7^a^	1.8 ± 0.1^b^	1.3 ± 1.1^c^
C20:5 *ω*-3 (EPA)	6.3 ± 0.5^a^	5.8 ± 0.3^b^	3.7 ± 0.7^c^
C22:2 *ω*-6	—	0.1 ± 0.8	—
C22:5 *ω*-3	—	—	—
C22:6 *ω*-3 (DHA)	5.1 ± 0.5^a^	6.1 ± 0.1^b^	6.0 ± 0.8^b^

∑PUFA	25.5	17.9	20.7

∑*ω*-3	13.4	13.2	10.7
∑*ω*-6	12.1	4.7	10.0
*ω*-3/*ω*-6	1.1	2.8	1.1

Values are reported as mean ± standard deviation.

Values in rows sharing same superscripts are not statistically different (*p* < 0.05).

—, not detected.

EPA: eicosapentaenoic acid; DHA: docosahexaenoic acid.

**Table 5 tab5:** Fatty acid composition of important freshwater fishes.

Fatty acids (%)	*C. catla*	*L. rohita*	*C. mrigala*	*S. seenghala* ^1^	*H. fossilis*	*C. batrachus*	*T. ilisha* ^2^	*R. rita*
*Saturated fatty acids (SFA)*								
C4:0-C5:0	—	—	—	—	—	—	—	—
C6:0	—	0.0 ± 0.0^a^	—	—	0.0 ± 0.0^a^	—	—	—
C8:0	—	—	0.0 ± 0.0^a^	0.0 ± 0.0^a^	0.0 ± 0.0^a^	—	0.0 ± 0.0^a^	—
C10:0	—	0.0 ± 0.0^a^	0.2 ± 0.0^b^	0.0 ± 0.0^a^	0.0 ± 0.0^a^	0.0 ± 0.0^a^	0.0 ± 0.0^a^	—
C11:0	—	0.0 ± 0.0^a^	1.1 ± 0.0^b^	—	0.0 ± 0.0^a^	0.0 ± 0.0^a^	—	—
C12:0	0.2 ± 0.0^a^	0.2 ± 0.0^a^	0.4 ± 0.1^b^	0.6 ± 0.1^c^	0.4 ± 0.0^b^	0.0 ± 0.6^d^	0.4 ± 0.2^b^	—
C13:0	0.2 ± 0.0^a^	0.3 ± 0.0^a^	0.4 ± 0.0^b^	0.1 ± 0.0^c^	0.1 ± 0.0^c^	0.0 ± 0.1^d^	0.0 ± 0.0^d^	1.5 ± 0.1^e^
C14:0	—	1.9 ± 0.3^a^	—	7.1 ± 2.1^b^	1.8 ± 0.3^a^	—	37.8 ± 0.2^c^	—
C15:0	—	—	7.4 ± 1.0^a^	2.6 ± 0.5^b^	1.1 ± 0.1^c^	0.4 ± 0.9^d^	1.5 ± 0.0^c^	0.2 ± 0.1^d^
C16:0	—	59.7 ± 9.8^a^	—	21.1 ± 3.2^b^	47.2 ± 7.5^c^	8.5 ± 2.3^d^	0.2 ± 0.0^e^	15.8 ± 2.9^f^
C17:0	—	1.9 ± 0.5^a^	4.4 ± 0.3^b^	2.7 ± 0.6^c^	0.5 ± 0.0^d^	0.6 ± 1.3^d^	1.0 ± 0.3^e^	0.4 ± 0.4^d^
C18:0	14.2 ± 4.6^a^	5.3 ± 1.2^b^	7.2 ± 2.9^c^	0.2 ± 0.0^d^	7.3 ± 1.3^c^	9.3 ± 2.5^e^	0.3 ± 0.0^d^	5.4 ± 1.1^e^
C20:0	0.5 ± 0.0^a^	0.2 ± 0.0^a^	0.7 ± 0.0^b^	0.7 ± 0.1^b^	0.0 ± 0.0^c^	—	—	—
C21:0	1.0 ± 0.2^a^	3.3 ± 0.9^b^	7.7 ± 1.1^c^	—	4.1 ± 1.2^d^	1.9 ± 1.3^a^	0.7 ± 0.1^e^	—
C22:0	0.9 ± 0.1^a^	0.2 ± 0.0^b^	0.5 ± 0.0^c^	0.3 ± 0.0^c^	0.1 ± 0.0^d^	—	0.4 ± 0.0^a^	—
C23:0	3.6 ± 1.0^a^	0.3 ± 0.0^b^	0.6 ± 0.2^c^	—	0.2 ± 0.0^b^	—	—	—
C24:0	—	—	—	0.4 ± 0.0^a^	—	—	0.4 ± 0.0^a^	—

∑SFA	20.6	73.3	30.4	36.0	63.1	21.0	42.8	23.3

*Monounsaturated fatty acids (MUFA)*								
C14:1	0.2 ± 0.0^a^	0.0 ± 0.0^b^	1.1 ± 0.0^c^	0.3 ± 0.0^a^	0.0 ± 0.0^b^	0.1 ± 0.2^d^	0.2 ± 0.0^a^	—
C15:1	—	0.0 ± 0.0^a^	—	0.1 ± 0.0^a^	2.4 ± 0.9^b^	0.5 ± 0.9^c^	0.0 ± 0.0^a^	—
C16:1	2.6 ± 0.9^a^	—	6.2 ± 0.3^b^	1.3 ± 0.1^c^	6.6 ± 1.6^b^	4.9 ± 1.6^c^	0.5 ± 0.0^d^	6.3 ± 0.9^b^
C17:1	2.7 ± 0.8^a^	0.5 ± 0.0^b^	2.7 ± 0.2^a^	1.9 ± 0.2^c^	0.1 ± 0.0^d^	—	0.3 ± 0.0^b^	—
C18:1	41.0 ± 8.9^a^	9.5 ± 2.3^b^	15.8 ± 6.9^c^	20.5 ± 3.9^d^	13.6 ± 5.4^e^	47.9 ± 5.6^f^	30.6 ± 0.2^g^	35.3 ± 0.0^h^
C20:1	0.8 ± 0.0^a^	0.3 ± 0.0^b^	0.0 ± 0.0^c^	2.0 ± 0.3^d^	—	—	2.3 ± 0.4^d^	20.6 ± 1.9^e^
C22:1	—	—	0.1 ± 0.5^a^	1.8 ± 0.2^b^	—	—	0.6 ± 0.0^c^	2.9 ± 0.2^d^
C24:1	—	—	—	0.5 ± 0.0^a^		—	0.8 ± 0.0^b^	—

∑MUFA	47.3	10.4	26.0	28.4	22.7	53.5	35.0	65.2

*Polyunsaturated fatty acids (PUFA)*								
C18:2 *ω*-6	6.7 ± 2.3^a^	7.6 ± 2.1^b^	14.0 ± 7.8^c^	4.5 ± 1.3^d^	5.8 ± 1.3^a^	22.2 ± 4.6^e^	2.7 ± 0.4^f^	0.2 ± 0.0^g^
C18:3 *ω*-3	10.9 ± 2.6^a^	6.3 ± 1.9^b^	3.3 ± 0.9^c^	4.7 ± 1.1^d^	3.3 ± 0.9^c^	1.5 ± 0.9^e^	2.2 ± 0.9^f^	0.7 ± 0.0^g^
C18:3 *ω*-6	—	0.2 ± 0.0^a^	0.5 ± 0.0^b^	1.4 ± 0.9^c^	0.1 ± 0.0^a^	0.5 ± 0.1^b^	0.7 ± 0.1^d^	—
C20:2 *ω*-6	0.7 ± 0.1^a^	0.0 ± 0.0^b^	0.0 ± 0.1^b^	1.3 ± 0.7^c^	0.0 ± 0.0^b^	—	0.1 ± 0.0^d^	—
C20:3 *ω*-6	1.4 ± 0.3^a^	0.6 ± 0.0^b^	3.4 ± 0.2^c^	2.2 ± 0.9^d^	0.6 ± 0.1^b^	0.7 ± 0.3^e^	0.1 ± 0.0^f^	—
C20:3 *ω*-3	0.2 ± 0.0^a^	0.1 ± 0.0^a^	3.1 ± 0.0^b^	1.0 ± 0.1^c^	0.4 ± 0.0^d^	—	0.2 ± 0.0^a^	—
C20:4 *ω*-6	0.5 ± 0.0^a^	6.3 ± 2.3^b^	17.6 ± 0.0^c^	9.8 ± 2.3^d^	0.2 ± 0.0^a^	—	4.1 ± 1.3^e^	1.6 ± 0.0^f^
C20:5 *ω*-3 (EPA)	6.8 ± 1.2^a^	0.9 ± 0.1^b^	1.5 ± 0.3^c^	4.4 ± 0.9^d^	1.5 ± 0.2^c^	—	2.9 ± 0.9^e^	3.8 ± 0.6^f^
C22:6 *ω*-3 (DHA)	4.7 ± 0.9^a^	0.4 ± 0.0^b^	—	6.2 ± 1.3^c^	2.2 ± 0.6^d^	0.5 ± 0.8^b^	8.9 ± 2.5^e^	5.0 ± 0.6^f^

∑PUFA	31.9	22.5	43.5	35.5	14.2	25.5	22.0	11.4

∑*ω*-3	22.7	7.8	7.8	16.3	7.3	2.8	14.2	9.5
∑*ω*-6	9.3	14.7	35.7	19.2	6.9	22.7	5.4	1.9
*ω*-3/*ω*-6	2.4	0.5	0.2	0.8	1.1	1.8	2.3	5.0

^1,2^Data previously published by Mohanty et al. [15, 31].

Values are reported as mean ± standard deviation.

Values in rows sharing same superscripts are not statistically different (*p* < 0.05).

—, not detected.

EPA: eicosapentaenoic acid; DHA: docosahexaenoic acid.

**Table 6 tab6:** Fatty acid composition of important small indigenous fishes.

Fatty acids (%)	*A. mola*	*P. sophore* ^1^	*A. coila*	*G. chapra*	*A. testudineus*	*X. cancila*
*Saturated fatty acid (SFA)*						
C4:0	—	—	—	—	—	—
C6:0	—	—	1.9 ± 0.1^a^	1.4 ± 0.1^a^	0.1 ± 0.0^b^	2.7 ± 0.1^c^
C7:0	—	—	0.0 ± 0.0^a^	—	—	1.2 ± 0.1^b^
C8:0	—	0.0 ± 0.0^a^	—	0.4 ± 0.1^b^	0.2 ± 0.0^b^	2.6 ± 0.2^c^
C9:0	—	—	2.3 ± 0.1^a^	2.5 ± 0.1^a^	—	4.1 ± 0.6^b^
C10:0	—	0.0 ± 0.0^a^	0.8 ± 0.0^b^	1.0 ± 0.2^c^	0.0 ± 0.0^a^	10.0 ± 0.9^d^
C11:0	—	—	1.3 ± 0.1^a^	1.3 ± 0.1^a^	0.0 ± 0.0^b^	6.0 ± 0.4^c^
C12:0	0.0 ± 0.0^a^	0.6 ± 0.1^b^	3.1 ± 0.3^c^	2.3 ± 0.1^d^	0.4 ± 0.0^b^	7.2 ± 0.2^e^
C13:0	0.0 ± 0.0^a^	0.2 ± 0.0^b^	2.2 ± 0.4^c^	1.1 ± 0.0^d^	0.1 ± 0.0^b^	4.0 ± 0.1^e^
C14:0	0.4 ± 0.0^a^	7.6 ± 1.5^b^	25.9 ± 1.2^c^	31.7 ± 0.9^d^	1.3 ± 0.4^e^	9.2 ± 0.6^b^
C15:0	0.1 ± 0.0^a^	3.4 ± 1.1^b^	10.7 ± 1.9^c^	8.4 ± 0.3^d^	1.3 ± 0.5^e^	6.3 ± 0.6^f^
C16:0	1.2 ± 0.1^a^	1.0 ± 0.1^a^	—	—	40.6 ± 9.8^b^	4.7 ± 0.4^c^
C17:0	—	4.2 ± 0.6^a^	0.0 ± 0.0^b^	—	2.7 ± 0.9^b^	3.6 ± 0.2^a^
C18:0	0.3 ± 0.0^a^	0.1 ± 0.0^a^	1.3 ± 0.1^a^	—	15.3 ± 4.5^c^	3.0 ± 0.3^d^
C19:0	1.6 ± 0.0^a^	—	0.0 ± 0.0^b^	—	—	1.6 ± 0.3^a^
C20:0	0.0 ± 0.0^a^	1.3 ± 0.5^b^	0.0 ± 0.0^a^	0.8 ± 0.0^c^	0.7 ± 0.1^c^	3.1 ± 0.8^d^
C21:0	0.0 ± 0.0^a^	1.0 ± 0.3^b^	0.0 ± 0.0^a^	0.1 ± 0.0^c^	3.2 ± 1.1^d^	3.4 ± 0.4^d^
C22:0	0.0 ± 0.0^a^	0.0 ± 0.0^a^	1.1 ± 0.1^b^	0.4 ± 0.0^c^	0.3 ± 0.0^c^	4.1 ± 0.7^d^
C23:0	0.0 ± 0.0^a^	—	0.3 ± 0.0^b^	0.0 ± 0.0^a^	—	4.9 ± 0.3^d^
C24:0	0.0 ± 0.0^a^	0.4 ± 0.0^b^	0.9 ± 0.0^c^	0.5 ± 0.0^b^	—	4.7 ± 0.5^d^

∑SFA	2.2	20.0	51.9	51.9	66.3	86.9

*Monounsaturated fatty acids (MUFA)*						
C14:1	0.3 ± 0.0^a^	0.3 ± 0.0^a^	3.3 ± 0.3^b^	1.2 ± 0.0^c^	—	2.0 ± 0.3^d^
C15:1	—	0.0 ± 0.0^a^	—	—	0.5 ± 0.1^b^	—
C16:1	5.2 ± 1.1^a^	4.4 ± 1.1^b^	0.4 ± 0.0^c^	—	8.6 ± 2.3^b^	1.7 ± 0.1^d^
C17:1	—	1.6 ± 0.2^a^	—	—	0.7 ± 0.1^b^	—
C18:1	64.3 ± 8.9^a^	28.6 ± 6.6^b^	—	1.9 ± 0.5^c^	—	2.4 ± 0.3^d^
C20:1	0.3 ± 0.0^a^	1.5 ± 0.2^b^	5.2 ± 0.9^c^	2.3 ± 0.5^d^	0.7 ± 0.1^e^	1.8 ± 0.9^b^
C22:1	—	0.1 ± 0.0^a^	—	—	—	0.0 ± 0.0^b^
C24:1	3.2 ± 1.1^a^	0.6 ± 0.0^b^	0.2 ± 0.1^c^	1.1 ± 0.3^d^	—	0.9 ± 0.0^d^

∑MUFA	73.3	37.1	9.1	6.5	10.4	9.1

*Polyunsaturated fatty acids (PUFA)*						
C18:2 *ω*-6	8.1 ± 2.4^a^	1.3 ± 0.3^b^	0.0 ± 0.0^c^	0.4 ± 0.0^d^	0.2 ± 0.0^d^	0.9 ± 0.0^b^
C18:3 *ω*-3	6.7 ± 1.3^a^	16.6 ± 5.6^c^	—	14.9 ± 1.2^b^	17.8 ± 6.7^c^	0.5 ± 0.0^d^
C18:3 *ω*-6	—	—	—	—	0.4 ± 0.0	—
C20:2 *ω*-6	—	2.0 ± 0.5	—	—	—	—
C20:3 *ω*-6	—	2.0 ± 0.3^a^	—	—	0.9 ± 0.1^b^	—
C20:3 *ω*-3	—	1.2 ± 0.6^a^	—	—	0.1 ± 0.0^b^	—
C20:4 *ω*-6	4.6 ± 1.6^a^	9.8 ± 2.3^b^	26.8 ± 0.3^c^	9.8 ± 0.6^b^	1.2 ± 0.4^d^	0.4 ± 0.0^d^
C20:5 *ω*-3 (EPA)	2.2 ± 0.9^a^	6.2 ± 1.2^b^	—	—	0.4 ± 0.0^c^	0.4 ± 0.0^c^
C22:2 *ω*-6	—	0.4 ± 0.0^a^	0.4 ± 0.0^a^	0.7 ± 0.0^b^	—	—
C22:6 *ω*-3 (DHA)	3.1 ± 1.1^a^	3.3 ± 1.1^a^	9.3 ± 0.1^b^	6.0 ± 0.5^c^	2.7 ± 0.6^d^	0.1 ± 0.0^e^

∑PUFA	24.8	42.9	36.6	31.8	23.7	2.6

∑*ω*-3	12.0	27.2	9.3	20.9	21	1.1
∑*ω*-6	12.7	15.6	27.7	10.9	2.7	1.4
*ω*-3/*ω*-6	0.9	1.7	0.3	1.9	7.9	0.7

^1^Data previously published by Mahanty et al. [16].

Values are reported as mean ± standard deviation.

Values in rows sharing same superscripts are not statistically different (*p* < 0.05).

—, not detected.

EPA: eicosapentaenoic acid; DHA: docosahexaenoic acid.

**Table 7 tab7:** Fatty acid composition of important cold water fishes.

Fatty acids (%)	*O. mykiss* ^1^	*T. putitora* ^1^	*S. richardsonii* ^1^	*N. hexagonolepis* ^1^	*C. carpio* ^1^
*Saturated fatty acid (SFA)*					
C4–C11	—	—	—	—	—
C12	0.6 ± 0.1^a^	0.5 ± 0.0^a^	0.0 ± 0.0^b^	2.5 ± 0.9^c^	0.2 ± 0.0^a^
C13	0.1 ± 0.0^a^	0.0 ± 0.0^b^	0.0 ± 0.0^b^	0.0 ± 0.0^b^	0.0 ± 0.0^b^
C14	3.5 ± 1.1^a^	5.0 ± 1.6^b^	7.6 ± 1.6^c^	4.7 ± 0.8^b^	2.3 ± 0.5^d^
C15	0.3 ± 0.0^a^	0.6 ± 0.0^b^	0.4 ± 0.0^a^	0.4 ± 0.1^a^	0.5 ± 0.0^b^
C16	21.8 ± 9.8^a^	31.6 ± 8.9^b^	26.1 ± 6.5^c^	29.8 ± 8.9^d^	35.2 ± 8.7^e^
C17	0.5 ± 0.0^a^	0.5 ± 0.0^a^	0.6 ± 0.0^a^	0.6 ± 0.0^a^	0.7 ± 0.0^b^
C18	7.6 ± 1.6^a^	9.6 ± 2.3^b^	7.5 ± 2.9^a^	5.9 ± 1.3^c^	6.7 ± 1.6^d^
C19	—	0.2 ± 0.0^a^	0.1 ± 0.0^a^	0.1 ± 0.0^a^	0.2 ± 0.0^a^
C20	—	4.5 ± 1.3^a^	—	0.3 ± 0.0^b^	0.4 ± 0.1^b^
C22	—	0.4 ± 0.0	—	—	—

∑SFA	34.5	53.0	42.5	44.3	46.2

*Monounsaturated fatty acids (MUFA)*					
C16:1	8.2 ± 1.6^a^	9.6 ± 2.2^a^	21.3 ± 8.9^c^	11.1 ± 5.6^d^	9.6 ± 1.6^a^
C17:1	0.2 ± 0.0^a^	—	0.0 ± 0.0^b^	—	—
C18:1	24.3 ± 6.5^a^	12.1 ± 4.5^b^	14.6 ± 5.6^c^	10.9 ± 3.4^b^	17.3 ± 5.6^d^
C20:1	1.2 ± 0.4^a^	5.6 ± 1.3^b^	1.2 ± 0.3^a^	1.6 ± 0.2^a^	3.9 ± 1.3^c^
C22:1	0.8 ± 0.1^a^	0.7 ± 0.1^a^	0.1 ± 0.0^b^	0.4 ± 0.0^c^	0.2 ± 0.0^b^

∑MUFA	34.7	28.1	37.3	23.9	31.0

*Polyunsaturated fatty acids (PUFA)*					
C18:2 *ω*-6	13.8 ± 3.2^a^	7.4 ± 1.3^b^	2.1 ± 0.9^c^	7.6 ± 1.9^b^	10.0 ± 2.9^d^
C18:3 *ω*-3	4.8 ± 1.2^a^	0.6 ± 0.0^b^	1.8 ± 0.6^c^	7.7 ± 2.5^d^	—
C18:3 *ω*-6	—	0.4 ± 0.0^a^	0.4 ± 0.0^a^	—	0.2 ± 0.0^b^
C18:4 *ω*-3	—	—	—	—	0.0 ± 0.0
C20:2 *ω*-6	0.8 ± 0.1	—	—	—	—
C20:3 *ω*-6	0.8 ± 0.1	—	—	—	—
C20:3 *ω*-3	—	0.5 ± 0.0^a^	1.0 ± 0.9^b^	0.5 ± 0.0^a^	1.4 ± 0.2^b^
C20:4 *ω*-6	2.4 ± 0.5^a^	1.8 ± 0.1^b^	0.8 ± 0.2^c^	2.7 ± 0.6^a^	3.6 ± 0.8^d^
C20:5 *ω*-3 (EPA)	2.3 ± 0.6^a^	4.7 ± 1.2^b^	9.6 ± 2.3^c^	7.4 ± 2.3^d^	—
C22:5 *ω*-3	—	—	—	—	3.2 ± 1.0
C22:6 *ω*-3 (DHA)	6.4 ± 1.6^a^	2.7 ± 0.9^b^	3.8 ± 1.2^c^	5.2 ± 1.1^d^	5.1 ± 1.9^d^

∑PUFA	31.4	18.3	19.4	31.2	23.7

∑*ω*-3	13.6	8.6	16.2	20.9	9.8
∑*ω*-6	17.8	9.7	3.2	10.3	13.9
*ω*-3/*ω*-6	0.9	0.9	4.9	2.0	0.7

^1^Data previously published by Sarma et al. [17].

Values are reported as mean ± standard deviation.

Values in rows sharing same superscripts are not statistically different (*p* < 0.05).

—, not detected.

EPA: eicosapentaenoic acid; DHA: docosahexaenoic acid.

**Table 8:** Fatty acid composition of prawns and edible molluscs (shellfishes).

Fatty acids (%)	Prawns			Molluscs	
	*M. rosenbergii*	*P. monodon*	*F. indicus*	*C. madrasensis*	*P. viridis*
*Saturated fatty acids (SFA)*					
C4:0–C11:0	—	—	—	—	—
C12:0	—	—	—	0.9 ± 0.1	—
C14:0	6.0 ± 1.2^a^	1.2 ± 0.4^b^	1.4 ± 0.3^b^	4.2 ± 0.9^c^	—
C15:0	—	1.2 ± 0.1^a^	1.1 ± 0.3^a^	1.1 ± 0.4^a^	—
C16:0	14.2 ± 3.2^a^	19.7 ± 1.8^b^	17.1 ± 2.6^c^	26.8 ± 6.5^d^	24.6 ± 5.9^d^
C17:0	—	2.9 ± 0.4^a^	1.6 ± 0.2^b^	2.3 ± 1.1^a^	0.7 ± 0.1^d^
C18:0	11.5 ± 2.3^a^	12.4 ± 1.4^a^	12.1 ± 1.4^a^	8.5 ± 1.3^b^	5.9 ± 1.5^c^
C20:0	—	0.5 ± 0.1^a^	0.3 ± 0.1^a^	0.7 ± 0.3^a^	0.6 ± 0.1^a^
C21:0	—	—	1.4 ± 2.6	—	—
C22:0	—	0.2 ± 0.1^a^	0.3 ± 0.2^a^	0.3 ± 0.1^a^	1.5 ± 0.3^b^
C24:0	—	0.4 ± 0.2^a^	0.1 ± 0.1^b^	2.2 ± 0.8^c^	1.7 ± 0.6^d^

∑SFA	35.2	39.1	35.4	47.1	34.9

*Monounsaturated fatty acids (MUFA)*					
C14:1	—	—	—	0.8 ± 0.2^a^	1.0 ± 0.4^b^
C16:1	7.6 ± 1.2^a^	4.2 ± 0.9^b^	2.2 ± 1.0^c^	6.1 ± 1.6^a^	2.2 ± 0.9^c^
C17:1	—	1.4 ± 0.3^a^	0.7 ± 0.3^b^	—	—
C18:1	19.1 ± 3.2^a^	16.3 ± 0.9^b^	12.6 ± 1.4^c^	9.9 ± 2.6^d^	15.4 ± 4.2^b^
C20:1	—	0.7 ± 0.1^a^	0.4 ± 0.1^a^	0.5 ± 0.2^a^	—
C22:1	—	—	—	5.2 ± 1.2^a^	3.1 ± 1.1^b^
C24:1	—	—	0.4 ± 0.2^a^	1.1 ± 0.5^b^	1.6 ± 0.3^c^

∑MUFA	29.6	22.7	16.5	23.8	23.4

*Polyunsaturated fatty acids (PUFA)*					
C18:2 *ω*-6	10.8 ± 2.3^a^	7.1 ± 1.9^b^	3.6 ± 1.9^c^	3.2 ± 0.9^c^	1.2 ± 0.6^d^
C18:3 *ω*-3	2.1 ± 0.9^a^	2.8 ± 0.6^a^	1.3 ± 2.4^b^	1 ± 0.1^b^	1.3 ± 0.7^b^
C18:3 *ω*-6	—	0.2 ± 0.0^a^	0.3 ± 0.2^a^	1.8 ± 0.3^c^	0.7 ± 0.1^b^
C18:4 *ω*-3	—	0.5 ± 0.3^a^	0.5 ± 0.1^a^	1.6 ± 0.6^b^	1.7 ± 0.6^b^
C20:2 *ω*-6	—	0.2 ± 0.0^a^	0.1 ± 0.1^b^	1.4 ± 0.5^c^	0.4 ± 0.1^a^
C20:3 *ω*-6	—	—	—	1.6 ± 0.6^a^	0.9 ± 0.2^b^
C20:4 *ω*-6	6.6 ± 1.3^a^	7.9 ± 1.2^b^	8.9 ± 1.5^c^	2.4 ± 0.9^d^	0.9 ± 0.3^e^
C20:5 *ω*-3 (EPA)	7.4 ± 2.1^a^	12.8 ± 1.5^b^	10.6 ± 3.0^c^	7.3 ± 2.1^a^	10.2 ± 4.5^c^
C22:2 *ω*-6	—	0.3 ± 0.2	—	—	—
C22:5 *ω*-3	2.0 ± 0.9^a^	—	—	1.0 ± 0.1^b^	1.6 ± 0.3^c^
C22:6 *ω*-3 (DHA)	—	6.4 ± 1.4^a^	10.0 ± 1.0^b^	7.4 ± 2.6^c^	9.5 ± 2.1^d^

∑PUFA	35.2	38.4	35.6	28.8	28.6

∑*ω*-3	11.6	22.0	22.0	18.3	22.6
∑*ω*-6	23.5	16.3	13.6	10.5	4.2
*ω*-3/*ω*-6	0.4	1.3	1.6	1.7	5.3

Values are reported as mean ± standard deviation.

Values in rows sharing same superscripts are not statistically different (*p* < 0.05).

—, not detected.

EPA: eicosapentaenoic acid; DHA: docosahexaenoic acid.

**Table 9:** DHA, EPA, and DHA + EPA (mg/100 g wet wt.) content of some important food fishes studied.

Fish species	DHA	EPA	DHA + EPA
	(mg/100 g wet wt.)		
*Ailia coila*	180.0 ± 5.0	—	180.0 ± 5.0
*Amblypharyngodon mola*	133.3 ± 6.8	94.6 ± 5.4	227.9 ± 10.6
*Cyprinus carpio*	152.1 ± 9.8	—	152.1 ± 9.8
*Crassostrea madrasensis*	383.4 ± 3.1	377.9 ± 3.0	761.3 ± 6.9
*Epinephelus *spp.	107.8 ± 0.8	47.8 ± 0.4	155.7 ± 1.2
*Etroplus suratensis*	186.6 ± 31.5	115.3 ± 26.5	301.9 ± 45.6
*Fenneropenaeus indicus*	80.6 ± 12.3	84.5 ± 25.1	165.1 ± 33.2
*Gudusia chapra*	342.0 ± 10.2	—	342.0 ± 10.2
*Katsuwonus pelamis*	—	104.8 ± 0.8	104.8 ± 0.8
*Lates calcarifer*	127.6 ± 15.6	155.2 ± 13.3	282.7 ± 25.5
*Leiognathus splendens*	226.2 ± 1.8	224.5 ± 1.8	450.7 ± 2.5
*Neolissochilus hexagonolepis*	210.1 ± 2.3	301.8 ± 6.5	414.2 ± 5.0
*Oncorhynchus mykiss*	224.6 ± 2.2	81.5 ± 1.5	306.1 ± 2.6
*Penaeus monodon*	54.1 ± 14.9	108.5 ± 17.6	162.6 ± 22.2
*Perna viridis*	158.9 ± 1.3	169.9 ± 1.4	328.8 ± 3.2
*Puntius sophore*	161.7 ± 7.6	303.8 ± 8.3	465.5 ± 9.8
*Schizothorax richardsonii*	93.3 ± 1.0	235.8 ± 4.0	337.7 ± 3.1
*Sardinella longiceps*	534.9 ± 4.3	937.9 ± 7.5	1472.9 ± 6.9
*Sperata seenghala*	49.6 ± 1.5	35.2 ± 1.0	84.8 ± 2.9
*Tenualosa ilisha*	934.5 ± 37.1	304.5 ± 14.0	1239.0 ± 25.2
*Tor putitora*	115.5 ± 12.2	201.9 ± 13.1	316.9 ± 13.1
*Trichiurus lepturus*	567.8 ± 4.5	203.1 ± 1.6	770.9 ± 5.6
*Xenentodon cancila*	70.0 ± 4.0	—	70.0 ± 4.0

—, not detected.

## References

[1] Abeywardena M. Y., Patten G. S. (2011). Role of *ω*3 long-chain polyunsaturated fatty acids in reducing cardio-metabolic risk factors. *Endocrine, Metabolic & Immune Disorders-Drug Targets*.

[2] Bang H. O., Dyerberg J., Hjøorne N. (1976). The composition of food consumed by Greenland Eskimos. *Acta Medica Scandinavica*.

[3] Kidd P. M. (2007). Omega-3 DHA and EPA for cognition, behavior, and mood: clinical findings and structural-functional synergies with cell membrane phospholipids. *Alternative Medicine Review*.

[4] Conor W. E. (2000). Importance of n-3 fatty acids in health and diseases. *American Journal of Clinical Nutrition*.

[5] Calder P. C. (2012). The role of marine omega-3 (*n*-3) fatty acids in inflammatory processes, atherosclerosis and plaque stability. *Molecular Nutrition and Food Research*.

[6] Hashimoto M., Katakura M., Tanabe Y. (2015). n-3 fatty acids effectively improve the reference memory-related learning ability associated with increased brain docosahexaenoic acid-derived docosanoids in aged rats. *Biochimica et Biophysica Acta (BBA)—Molecular and Cell Biology of Lipids*.

[7] IFFO The importance of dietary EPA & DHA omega-3 fatty acids in the health of both animals and humans. http://www.iffo.net/system/files/75_0.pdf.

[8] Beare-Rogers J., Ghafoorunissa A., Korver O., Rocquelin G., Sundram K., Uauy R. (1998). Dietary fat in developing countries. *Food and Nutrition Bulletin*.

[9] Michaelsen K. F., Dewey K. G., Perez-Exposito A. B., Nurhasan M., Lauritzen L., Roos N. (2011). Food sources and intake of n-6 and n-3 fatty acids in low-income countries with emphasis on infants, young children (6–24 months), and pregnant and lactating women. *Maternal & Child Nutrition*.

[10] Abd Aziz N., Azlan A., Ismail A., Mohd Alinafiah S., Razman M. R. (2013). Quantitative determination of fatty acids in marine fish and shellfish from warm water of straits of malacca for nutraceutical purposes. *BioMed Research International*.

[11] Mohanty B. P., Ayyappan S., Moza U., Gopalakrishnan A. (2010). Fish as health food. *Handbook of Fisheries and Aquaculture*.

[12] AOAC (2000). *Official Methods of Analysis*.

[13] Folch J., Lees M., Sloane Stanley G. H. (1957). A simple method for the isolation and purification of total lipides from animal tissues. *The Journal of Biological Chemistry*.

[14] Metcalfe L. D., Schmitz A. A., Pelka J. R. (1966). Rapid preparation of fatty acid esters from lipids for gas chromatographic analysis. *Analytical Chemistry*.

[15] Mohanty B. P., Paria P., Mahanty A. (2012). Fatty acid profile of Indian shad *Tenualosa ilisha* oil and its dietary significance. *National Academy Science Letters*.

[16] Mahanty A., Ganguly S., Verma A. (2014). Nutrient profile of small indigenous fish *Puntius sophore*: proximate composition, amino acid, fatty acid and micronutrient profiles. *National Academy Science Letters*.

[17] Sarma D., Akhtar M. S., Das P. (2013). Nutritional quality in terms of amino acid and fatty acid of five coldwater fish species: implications to human health. *National Academy Science Letters*.

[18] US Department of Agriculture (2010). *Dietary Guidelines for Americans*.

[19] McKenney J. M., Sica D. (2007). Role of prescription omega-3 fatty acids in the treatment of hypertriglyceridemia. *Pharmacotherapy*.

[20] Mennitti L. V., Oliveira J. L., Morais C. A. (2015). Type of fatty acids in maternal diets during pregnancy and/or lactation and metabolic consequences of the offspring. *Journal of Nutritional Biochemistry*.

[21] Mohanty B. P., Ganguly S., Karunakaran D. (2012). Maternal fish consumption and prevention of low birth weight in the developing world. *National Academy Science Letters*.

[22] Holub B. J. (2009). Docosahexaenoic acid (DHA) and cardiovascular disease risk factors. *Prostaglandins Leukotrienes and Essential Fatty Acids*.

[23] Feldman H. M., Reiff M. I. (2014). Attention deficit-hyperactivity disorder in children and adolescents. *The New England Journal of Medicine*.

[24] Hoffman D. R., Boettcher J. A., Diersen-Schade D. A. (2009). Toward optimizing vision and cognition in term infants by dietary docosahexaenoic and arachidonic acid supplementation: a review of randomized controlled trials. *Prostaglandins, Leukotrienes and Essential Fatty Acids*.

[25] Alam A. K. M. N., Mohanty B. P., Hoq M. E., Thilsted S. H. Nutritional values, consumption and utilization of hilsa *Tenualosa ilisha* (Ham.).

[26] Burdock G. A., Carabin I. G. (2007). Safety assessment of myristic acid as a food ingredient. *Food and Chemical Toxicology*.

[27] Blanchet C., Lucas M., Julien P., Morin R., Gingras S., Dewailly É. (2005). Fatty acid composition of wild and farmed Atlantic salmon (*Salmo salar*) and rainbow trout (*Oncorhynchus mykiss*). *Lipids*.

[28] Mohanty B., Mahanty A., Ganguly S. (2014). Amino acid compositions of 27 food fishes and their importance in clinical nutrition. *Journal of Amino Acids*.

[29] Atanasoff A., Nikolov G., Staykov Y., Zhelyazkov G., Sirakov I. (2013). Proximate and mineral analysis of Atlantic salmon (*Salmo Salar)* cultivated in Bulgaria. *Biotechnology in Animal Husbandry*.

[30] Aneesh P. A., Varkey J., Anandan R. (2012). Omega-3 polyunsaturated fatty acid profile of four Indian food fishes of Arabian Sea. *Nutritional Medicine Health and Wellness*.

[31] Mohanty B. P., Paria P., Das D. (2012). Nutrient profile of giant river-catfish *Sperata seenghala* (Sykes). *National Academy Science Letters*.

